# Meclizine Prevents Ovariectomy-Induced Bone Loss and Inhibits Osteoclastogenesis Partially by Upregulating PXR

**DOI:** 10.3389/fphar.2017.00693

**Published:** 2017-10-04

**Authors:** Jiachao Guo, Weijin Li, Yingxing Wu, Xingzhi Jing, Junming Huang, Jiaming Zhang, Wei Xiang, Ranyue Ren, Zhengtao Lv, Jun Xiao, Fengjing Guo

**Affiliations:** ^1^Department of Orthopedics, Tongji Hospital, Tongji Medical College, Huazhong University of Science and Technology, Wuhan, China; ^2^Department of Otolaryngology-Head and Neck Surgery, Tongji Hospital, Tongji Medical College, Huazhong University of Science and Technology, Wuhan, China

**Keywords:** meclizine, PXR, osteoclast, RANKL, osteoporosis

## Abstract

Pregnane X receptor (PXR) which belongs to the nuclear hormone receptor superfamily plays vital roles in several biological functions, especially in the inflammatory procedure. Besides that, PXR is revealed by recent studies to have essential effects on bone tissue. As an agonist of PXR, meclizine is a piperazine-derived histamine H1 antagonist, and has been frequently used for prevention and treatment of vomiting and nausea. Because osteoclastogenesis is characterized by the activation of inflammation-related signaling pathways, we speculated that meclizine may affect formation and function of osteoclast. In the present study, we explored the effect of meclizine on RANKL-induced osteoclastogenesis both *in vivo* and *in vitro*. In primary bone marrow-derived macrophages (BMMs), meclizine reduced osteoclast formation and bone resorption in a dose-dependent manner, while knockdown of PXR with siRNA partially abrogated the osteoclastogenesis inhibition of meclizine. On the one hand, at the molecular level, meclizine attenuated RANKL-induced activation of c-Fos, NFATc1, nuclear factor-κB (NF-κB) and mitogen-activated protein kinase (MAPKs), including ERK and p38, but not JNK. Meanwhile, meclizine reduced the expression of osteoclast-specific genes, including *TRAP, MMP9, Cathepsin K* and *NFATc1*. On the other hand, meclizine decreased OVX-induced bone loss by repressing osteoclast activity. In conclusion, our results indicated that meclizine inhibits osteoclastogenesis via regulation of several RANKL signaling pathways and PXR was involved in the processes. Therefore, meclizine may be considered as a novel therapeutic candidate for osteoclast-related diseases.

## Introdution

Bone homeostasis constantly undergoes remodeling including bone resorption by osteoclasts and bone formation by osteoblasts (Boyle et al., 2003). Osteoclasts are multinucleated giant cells originated from hematopoietic stem cells, and possess main ability to resorb bones (Kikuta and Ishii, 2013). Excessive osteoclast-mediated bone resorption influences the balance of bone remodeling process in numerous bone metabolism diseases including rheumatoid arthritis, Paget’s disease, and postmenopausal osteoporosis (Takayanagi, 2007a; Novack and Teitelbaum, 2008). Therefore, one of the most valid strategies for treating osteoclast-related diseases is to aim at inhibition of genesis and activity of osteoclasts. Osteoclasts differentiation and function are chiefly regulated by 2 key cytokines, macrophage colony-stimulating factor (M-CSF) and receptor activator of nuclear factor-κB (NF-κB) ligand (RANKL). M-CSF stimulates survival and proliferation of osteoclast precursors by activating PI3K/AKT and ERK (Karsenty and Wagner, 2002). As for RANKL which belongs to the tumor necrosis factor family, it promotes osteoclast precursors differentiation into mature osteoclasts (Kong et al., 1999). Briefly, RANKL binds to RANK on osteoclast precursor cells, recruiting and accumulating the adaptor molecules, especially TRAF6 (Nakashima et al., 2011). TRAF6 subsequently induces downstream signaling pathways including NF-κB, MAPKs (ERK, p38 and JNK), NFATc1, and AP-1 (Lee et al., 2001). NFATc1 and c-fos are the prime factors of osteoclastogenesis. These transcription factors can activate the expression of osteoclastogenesis target genes, such as *MMP9, TRAP* and *cathepsin K*, which leads to enhance the differentiation and function of osteoclasts (Takayanagi, 2007b; Conaway et al., 2016). Interfering with these signaling pathways can help prevent and treat pathological bone loss.

The pregnane X receptor (PXR) belongs to the nuclear hormone receptor superfamily (Kliewer et al., 1998). Previous studies have indicated that PXR is crucial to xenobiotic metabolism in humans, rats, rabbits, and mice (Jones et al., 2000; Francis et al., 2003; Chirulli et al., 2005), and PXR also has been confirmed to play a vital role in endobiotic metabolism in humans, rats, as well as mice (Xie et al., 2001; Bachmann et al., 2004; Matic et al., 2007; Cho et al., 2009). The mouse PXR (mPXR) was first detected in 1998, and was found to be motivated by a variety of compounds, such as antifungals, steroids, pregnane derivatives, and herbal extracts (Blumberg et al., 1998; Kliewer et al., 1998; Jones et al., 2000; Moore et al., 2002). The human PXR (hPXR) ortholog have been shown to be the steroid and xenobiotic receptor (SXR) and pregnane activated receptor (PAR), both revealing structural features and activation patterns similar to mPXR (Blumberg et al., 1998; Chirulli et al., 2005). SXR/PAR also was subsequently identified to be orthologous to mPXR via the PXR knockout mice research (Xie et al., 2000; Chirulli et al., 2005). PXR is predominantly expressed in intestine and liver (Ma et al., 2008). Besides, it was detected in other organizations, including brain, heart, stomach, and peripheral mononuclear as well as immune cells (Staudinger et al., 2001; Lamba et al., 2004; Owen et al., 2004). Nevertheless, the functions of PXR in these cells require further investigation. Recent study indicates that PXR has essential effects on bone tissue (Azuma et al., 2014). Systemic deletion of PXR induced osteopenia with mechanical frangibility (Azuma et al., 2010), and PXR was detected to activate the expression of osteoblast marker genes (Tabb et al., 2003). These evidences demonstrate that PXR is beneficial for preventing bone loss.

Meclizine, a piperazine-derived histamine H1 antagonist, currently is used to treat for vertigo and motion sickness (Wang et al., 2012). Intriguingly, *in vivo* researches have demonstrated that meclizine has other pharmacological effects, including neuroprotective effects in Huntington’s disease models (Gohil et al., 2011), cardiac protection and neuroprotective effects against stroke (Gohil et al., 2010), and skeletal growth in transgenic mice with achondroplasia (Matsushita et al., 2015). Previously, meclizine was identified as an agonist of human PXR (Lau et al., 2011). Consequently, we hypothesized that meclizine may be a potential inhibitor of osteoclastogenesis. In this study, we explored the function of PXR in osteoclast formation and the effects of meclizine on RANKL-induced osteoclastogenesis using *in vivo* as well as *in vitro* models, and elucidated the underlying molecular mechanisms.

## Results

### Blocking of PXR Promotes Osteoclast Formation

**FIGURE 1:**
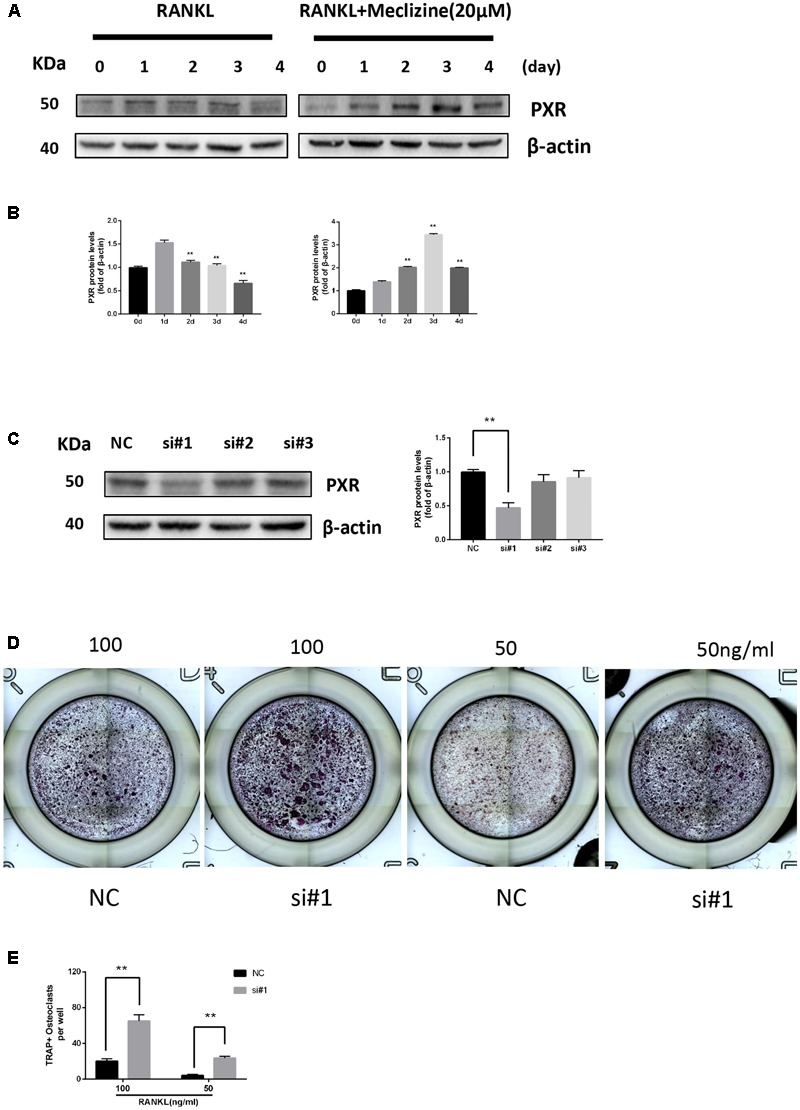
Pregnane X receptor (PXR) is repressed during osteoclastogenesis and upregulated by meclizine. PXR knockdown increases osteoclast differentiation. **(A,B)** BMMs were seeded on day 0, and cultured in the presence of RANKL (100 ng/mL) and M-CSF (30 ng/mL) with or without meclizine. Cells were collected to analyze PXR protein expression and β-actin was used as a loading control. Data are presented as mean ± SD of 3 independent experiments. ^∗∗^*P* < 0.01 versus 1 day group. **(C)** The expression of PXR in BMMs was evaluated by western blot analysis. Data are presented as mean ± SD of 3 independent experiments. ^∗∗^*P* < 0.01. **(D,E)** BMMs (1.0 × 10^4^ cells/well) were seeded in 96-well plates, transfected with PXR siRNA and cultured with M-CSF (30 ng/mL) and different concentrations of RANKL (50, 100 ng/mL) for 3 days. Subsequently, TRAP staining was performed, and TRAP-positive multinucleated cells (≥3 nuclei) were counted and photographed by a microscope. Data are presented as mean ± SD of 3 independent experiments. ^∗∗^*P* < 0.01.

We explored the role of PXR in osteoclast differentiation. The protein expression of PXR gradually decreased during RANKL-induced osteoclastogenesis in BMMs. However, PXR expression prominently increased and peaked on day 3 due to the influence of meclizine over time (**Figures 1A,B**). Next, PXR siRNA was transfected into BMMs (**Figure 1C**). In the presence of 100 ng/ml RANKL, it is observed that there is a significant promotion in osteoclastogenesis. In addition, the results showed that the transfected cells also increased TRAP+ multinuclear osteoclast formation when the concentration of RANKL decreased to 50 ng/ml (**Figures 1D,E**).

### Meclizine Represses RANKL-Induced Osteoclastogenesis *In Vitro*

**FIGURE 2:**
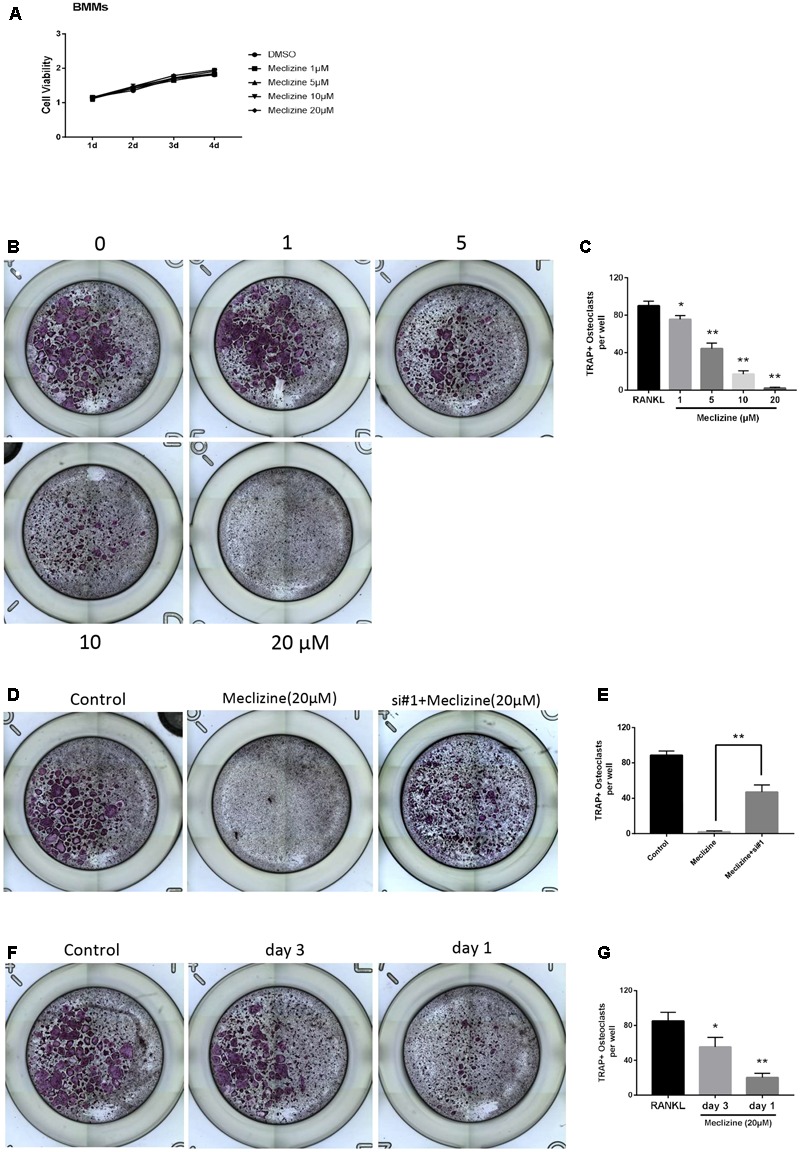
Meclizine inhibits RANKL-induced osteoclast formation. **(A)** Meclizine has a tiny effect on proliferation of BMMs. BMMs (6 × 10^3^ cells/well) were cultured with M-CSF (30 ng/mL) and various doses of meclizine (0, 1, 5, 10, and 20 μM) for 4 days. Data are presented as mean ± SD of 3 independent experiments. **(B,C)** Meclizine inhibits osteoclastogenesis in a dose-dependent manner. BMMs (1.0 × 10^4^ cells/well) were treated with different concentrations of meclizine every day in the presence of RANKL (100 ng/mL) and M-CSF (30 ng/mL) for 4 days. Then, the cells were stained for TRAP assay. TRAP-positive multinucleated osteoclasts (≥3 nuclei) were counted. Data are presented as means ± SD of three independent experiments. ^∗^*P* < 0.05, ^∗∗^*P* < 0.01 versus RANKL group. **(D,E)** BMMs (1.0 × 10^4^ cells/well) were incubated with RANKL (100 ng/mL), M-CSF (30 ng/mL), meclizine (20 μM) with or without PXR siRNA. Cells were used for TRAP staining after 4 days, and TRAP-positive multinucleated osteoclasts (≥3 nuclei) were counted. Data are presented as means ± SD of 3 independent experiments; ^∗∗^*P*<0.01. **(F,G)** BMMs (1.0 × 10^4^ cells/well) were cultured in the presence of RANKL (100 ng/mL) and M-CSF (30 ng/mL) for 4 days and treated with meclizine (20 μM) at the indicated times. Subsequently, TRAP staining was performed, and TRAP-positive osteoclasts (≥3 nuclei) were counted. Data are presented as means ± SD of 3 independent experiments; ^∗^*P* < 0.05, ^∗∗^*P* < 0.01 versus RANKL group.

The TRAP staining results indicated that meclizine inhibited the formation of osteoclasts in a dose-dependent manner at 1–20 μM (**Figures 2B,C**). At the concentration of 20 μM meclizine, there were no visible TRAP-positive multinucleated cells. However, the maximal inhibitory effect of meclizine observed at 20 μM was reversed upon treatment with PXR siRNA (**Figures 2D,E**). As shown in **Figure 2A**, the CCK8 outcomes demonstrated that 1–20 μM meclizine did not impact the viability of BMMs. Moreover, meclizine was added at diverse time points during osteoclast differentiation to evaluate which differentiation stage was affected. The results revealed that meclizine suppressed the quantity of TRAP+ multinuclear cells at both early and later stages (**Figures 2F,G**).

### Meclizine Inhibits Osteoclast Function

**FIGURE 3:**
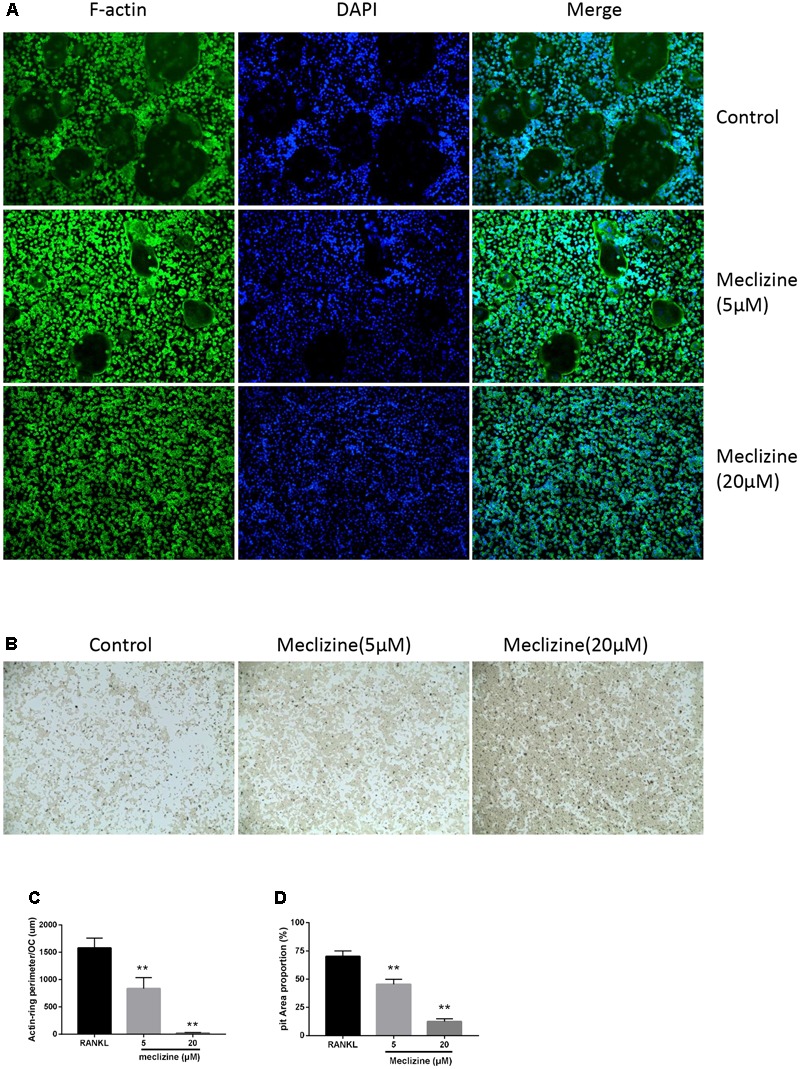
Meclizine suppresses actin ring formation and osteoclast function. **(A,C)** Meclizine disrupts actin ring formations. Staining for actin ring formation was performed after culturing for 4 days. Typically fluorescence microscopy images of BMMs (1.0 × 10^4^ cells/well) treated with meclizine (0, 5, 20 μM) are shown and the perimeters of actin rings were measured. Data are presented as means ± SD. ^∗∗^*P* < 0.01 versus RANKL group. **(B,D)** Meclizine inhibited the bone resorption activity of osteoclasts. BMMs (2.0 × 10^4^ cells/well) seeded onto a Corning Osteo Assay Surface were treated with RANKL (100 ng/mL) and M-CSF (30 ng/mL) for 4 days and subsequently treated with 0, 5 or 20 μM meclizine for an additional 4 days. Images of erosion pits were taken and the percentage of erosion area (blank area)/total area was determined. Data are presented as means ± SD of 3 independent experiments; ^∗∗^*P* < 0.01 versus RANKL group.

To further examine the influence of meclizine on the function of mature osteoclasts, actin ring formation assays and bone slice resorption assays were performed. Actin ring plays pivotal roles in osteoclast attachment and bone resorption, and the results indicated that actin ring formation was inhibited by meclizine treatment (**Figures 3A,C**). Furthermore, BMMs were cultured with RANKL (100 ng/mL) and M-CSF (30 ng/mL) for 4 days after seeding onto a bone slice, and then additional 4 days in the presence of different concentrations of meclizine. Finally, the resorption pits were quantified. As shown in **Figures 3B,D**, meclizine signally repressed the bone resorption function of osteoclasts. These findings indicated that meclizine impairs the function of mature osteoclasts.

### Meclizine Represses OVX-Induced Bone Loss and Osteoclast Activity

**FIGURE 4:**
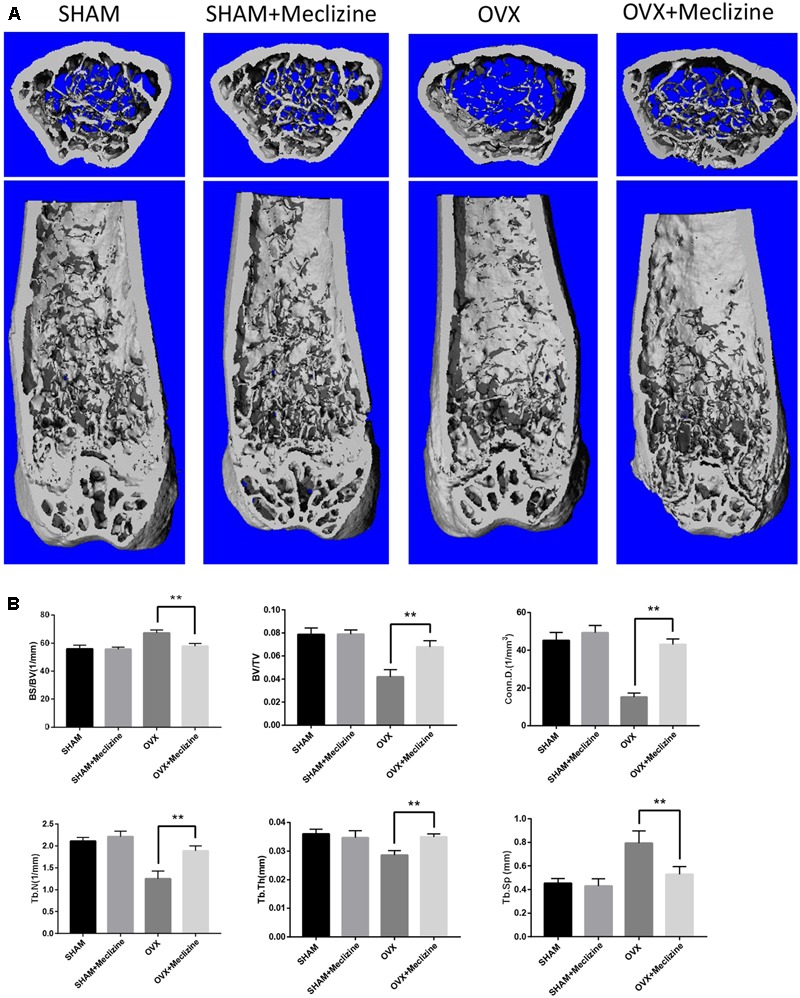
Meclizine prevents bone loss in OVX mice. **(A)** Micro-CT images of the distal femoral metaphyseal region from the SHAM, SHAM + meclizine, OVX, and OVX + meclizine groups. **(B)** Histograms represent the trabecular structural parameters of the distal femur: bone surface/trabecular bone volume (BS/BV), trabecular bone volume/tissue volume (BV/TV), connectivity density (Conn.D), trabecular number (Tb.N), trabecular thickness (Tb.Th) and trabecular separation (Tb.Sp). Data are presented as means ± SD. *n* = 10. ^∗∗^*P* < 0.01.

**FIGURE 5:**
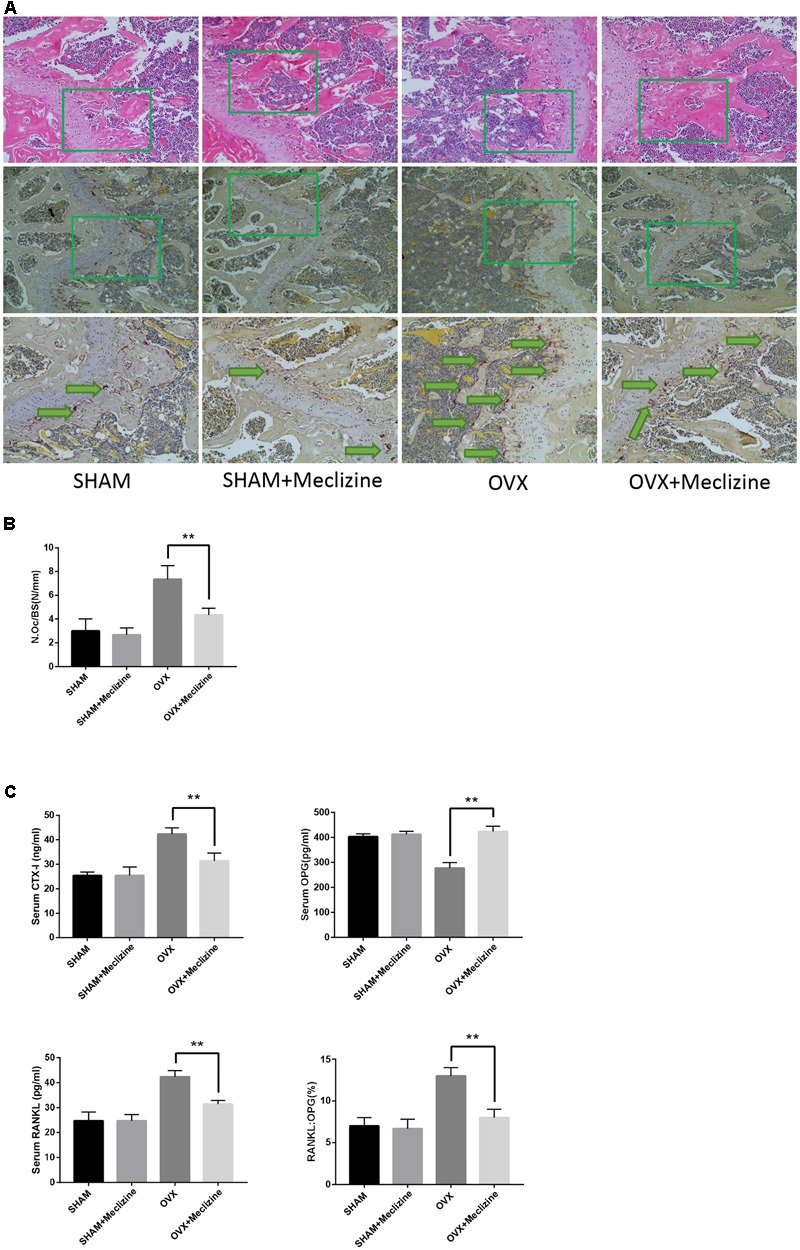
Meclizine reduces OVX-induced osteoclast formation and decreases the serum levels of CTX-I, OPG and RANKL. **(A)** The paraffin-embedded bone sections of distal femurs were stained for H&E and TRAP. **(B)** The osteoclast number/bone surface was quantified. Data represent as mean ± SD. *n* = 10. ^∗∗^*P* < 0.01. **(C)** Serum levels of CTX-I, OPG, and RANKL were determined by ELISA. Data are presented as mean ± SD. *n* = 10. ^∗∗^*P* < 0.01.

We used the OVX mice model to simulate osteoporosis in postmenopausal women. Meclizine (20 mg/kg) exhibited no influence on body weight of mice that was recorded every 2 weeks (Supplementary Figure 1A). Next, micro-CT was used to analyze the trabecular bone changes in distal femur of different model groups. OVX + meclizine group demonstrated dramatically increase in BV/TV, Tb.N, Tb.Th, Conn.D, while decrease in Tb.Sp and BS/BV when compared with the OVX group. In short, OVX + meclizine group significantly attenuated trabecular bone loss, and there was no significant difference between SHAM + meclizine group and SHAM group (**Figures 4A,B**). Moreover, the OVX and OVX + meclizine groups showed obviously reduced wet weight of uterus compared with the SHAM and SHAM + meclizine group (Supplementary Figure 1B), suggesting the success of ovariectomy. Hematoxylin and eosin (H&E) staining of the decalcified femurs were used to further confirm our results (**Figure 5A**). The trabeculae in the OVX group were rare both proximally and distally to the growth plate. OVX + meclizine group observably increased trabecular density and thickness when compared with the OVX group.

Next, TRAP staining was performed on the femoral sections. The size and activity of osteoclasts in OVX group significantly increased compared with SHAM and SHAM + meclizine groups. N.Oc/BS, the osteoclastic parameters, confirmed that OVX + meclizine group generated signally reduced numbers of TRAP-positive multinucleated cells at the growth plates of the femur when compared with the OVX group (**Figures 5A,B**).

In addition, the serum levels of CTX-I and RANKL induced by OVX were significantly decreased in OVX + meclizine group (**Figure 5C**). Whereas, serum OPG levels were markedly increased by meclizine treatment, and the RANKL/OPG ratio in OVX + meclizine group obviously decreased when compared with OVX group (**Figure 5C**). Altogether these observations suggest that meclizine can attenuate OVX-induced bone loss as a strong inhibitor of osteoclastogenesis and resorption activity.

### Meclizine Represses Expression of Osteoclast-Related Genes

**FIGURE 6:**
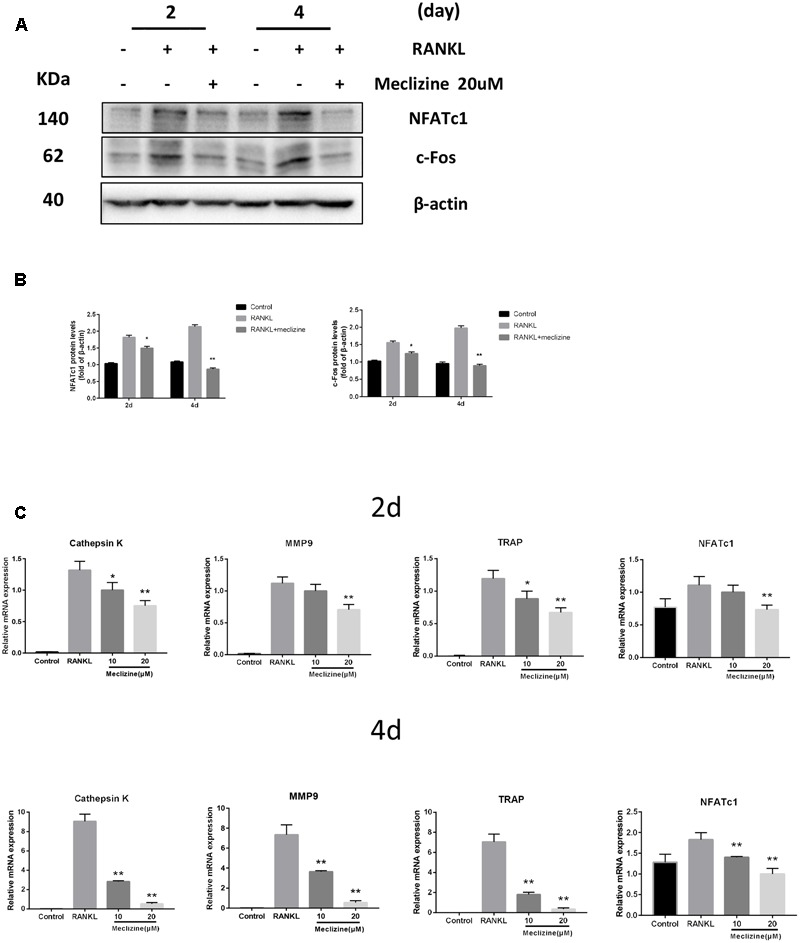
Meclizine decreases expression of osteoclast maker genes in BMMs. **(A,B)** Meclizine inhibits RANKL-induced protein expression of NFATc1 and c-Fos. BMMs were treated with or without meclizine (20 μM) in the presence of RANKL (100 ng/mL) and M-CSF (30 ng/ml). Protein expression levels were measured by western blotting at the indicated times and β-actin was used as a loading control. The experiments were repeated three times independently; ^∗^*P* < 0.05, ^∗∗^*P* < 0.01 versus RANKL group. **(C)** Meclizine represses RANKL-induced mRNA expression of *MMP9, Cathepsin K, TRAP* and *NFATc1*. BMMs were treated with or without meclizine (20 μM) in the presence of RANKL (100 ng/mL) and M-CSF (30 ng/ml) for 2 or 4 days. Total RNA was extracted and mRNA expression was determined by quantitative real-time RT-PCR. Data are represented as mean ± SD. ^∗^*P* < 0.05; ^∗∗^*P* < 0.01 versus RANKL group.

NFATc1 is the chief transcription factor for osteoclast function and differentiation (Takayanagi et al., 2002), which is in part regulated by c-Fos during osteoclastogenesis (Matsuo et al., 2004). Several osteoclast marker genes including *TRAP, Cathepsin K, NFATc1* and *MMP9* are target genes of NFATc1 (Asagiri and Takayanagi, 2007). **Figures 6A,B** revealed that meclizine obviously inhibited the expression of NFATc1 and c-Fos which are RANKL-induced. Meanwhile, this study also explored whether meclizine inhibited the mRNA expression of the osteoclast-specific genes. The findings demonstrated that meclizine observably suppresses the expression level of *TRAP, Cathepsin K, NFATc1* and *MMP9* at both early and late stage of osteoclastogenesis (**Figure 6C**).

### Meclizine Suppresses RANKL-Induced NF-κB and MAPKs Activation

**FIGURE 7:**
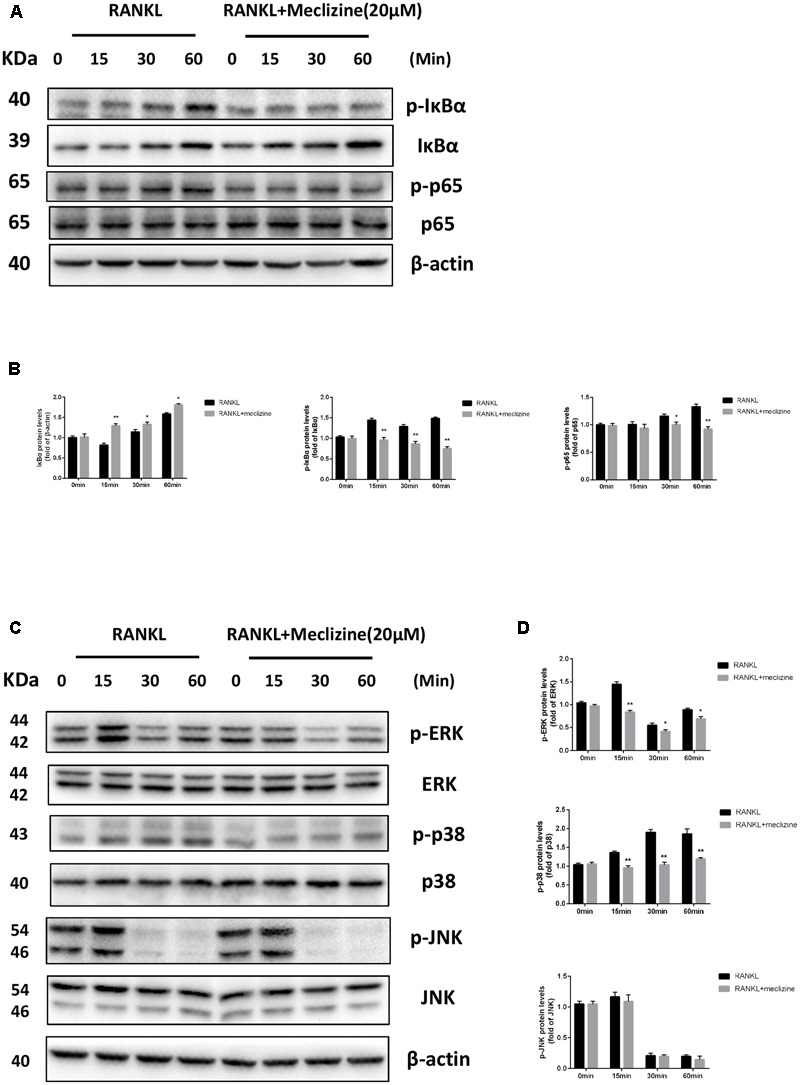
Meclizine represses RANKL-induced NF-κB and MAPKs in BMMs. **(A,B)** Meclizine inhibits NF-κB activation. BMMs were starved with α-MEM in the absence of FBS for 12 h, and then pretreated with or without meclizine (20 μM) for 1 h. Subsequently, BMMs were treated with or without RANKL (100 ng/mL) for the indicated times. The total proteins were extracted for immunoblotting with the indicated antibodies. **(C,D)** The effect of meclizine on RANKL-induced phosphorylation of ERK, p38 and JNK. BMMs were treated as described above, and total proteins were extracted for immunoblotting with MAPKs antibodies. The experiments were repeated three times independently and the total protein was used as a loading control. ^∗^*P* < 0.05, ^∗∗^*P* < 0.01 versus RANKL group.

Activation of NF-κB and MAPKs plays essential roles in RANKL-mediated osteoclastogenesis. Therefore, further analyses were carried out to explore whether the effect of meclizine inhibits osteoclast differentiation through these pathways. As shown in **Figures 7A,B**, because of NF-κB activation, RANKL induced IκB-α degradation at 15 min and IκB-α resynthesis at 30 min. Meclizine signally suppressed RANKL-induced degradation and phosphorylation of IκB-α, and the phosphorylation of NF-κB p65. As for MAPKs, meclizine inhibits phosphorylation of ERK and p38, but did not affect p-JNK levels during osteoclastogenesis (**Figures 7C,D**).

## Discussion

In the current study, results demonstrated that meclizine effectively restrained bone loss and suppressed osteoclastogenesis both *in vivo* and *vitro*. To our knowledge, the effect of meclizine on bone metabolism was explored for the first time. We confirmed that meclizine impaired osteoclast function and actin ring formation and efficiently repressed multiple pathways downstream of RANK at the molecular level, including NF-κB, MAPKs, NFATc1 and AP-1. In addition, meclizine can also decrease the serum levels of CTX-I and RANK: OPG ratio, which have critical regulatory influences on osteoclastogenesis.

We investigated that PXR protein expression was decreased during RANKL-induced osteoclastogenesis and knockdown of PXR enhanced osteoclastogenesis. Recent study demonstrated that PXR knockout mice display osteopenia. All in all, our experiments about PXR *in vitro* are consistent with previous findings. Meclizine used to be identified as an agonist of human PXR which binds directly to the human PXR ligand-binding domain; it also promotes the protein expression of mouse PXR which has similar structural features and activation patterns to human PXR. Moreover, PXR siRNA can abolish the inhibition of meclizine on osteoclastogenesis. These results indicated that PXR could be an essential factor mediating the function of meclizine in the process.

Previous studies of the PXR and the inflammatory vital mediator NF-κB have demonstrated a functional link between xenobiotic neutralization and inflammation that elucidated how certain xenobiotics affects the immune response (Zhou et al., 2006). NF-κB, a pivotal regulator of osteoclast function and formation (Takayanagi, 2007a), is one of the most significant downstream pathways mediating the function of meclizine on osteoclast differentiation via PXR. Our results revealed that meclizine inhibited the phosphorylation of IκB-α and p65. This is in agreement with previous studies, which have confirmed that PXR intervenes in a series of inflammatory diseases through the NF-κB pathway and inhibition of NF-κB suppressed osteoclast formation associated with inflammation *in vivo* (Jimi et al., 2004; Shah et al., 2006; Mencarelli et al., 2011; Dou et al., 2013; Zhang J. et al., 2015).

MAPKs mainly include p38, ERK and JNK. These proteins are stimulated by RANKL and have been found to be involved in osteoclastogenesis. Activation of AP-1 initiated by MAPKs also facilitates the induction and further auto-amplification of NFATc1 (Gohda et al., 2005). We detected that meclizine markedly inhibited phosphorylation of ERK and p38 triggered by RANKL. These results were in line with previous studies, suggesting that meclizine attenuates ERK phosphorylation in chondrocytes and rifaximin acts as a PXR agonist similar to meclizine significantly blocked p38 phosphorylation (Matsushita et al., 2013; Esposito et al., 2016). Moreover, our data showed that meclizine obviously repressed RANKL-induced expression of NFATc1 and c-Fos, and subsequently inhibited the activation of osteoclast-derived marker genes, including *cathepsin K* and *MMP9*, which can straightly degrade collagens in hard tissues (Takayanagi, 2007b). Taken together, these findings suggest that multiple signals interact with meclizine to inhibit osteoclast differentiation and activity.

Despite the findings discovered by this study, its limitations cannot be ignored. The homeostasis of bone comes from the balance of osteoclast and osteoblast activity. Our results illustrated that meclizine prevented OVX-induced bone loss *in vivo*, but whether meclizine promotes osteoblastogenesis remains to be proven. As the main mediate factor of meclizine, the concrete mechanism about PXR on differentiation and function in osteoclasts need to be further studied.

Meclizine has been shown to be beneficial in some disease researches, including Parkinson disease (Hong et al., 2016), achondroplasia (Matsushita et al., 2015), and colon cancer (Lin et al., 2007). In conclusion, our study demonstrated that meclizine has a significant inhibitory effect on osteoclastogenesis and OVX-induced bone loss. These results suggested that meclizine may serve as a latent therapeutic strategy for osteoclast-related disorders. In consideration of, in view of the increase of the population aging and the demand of osteoporosis treatment as well as the shortcomings of current anti-osteoporotic drugs, it will be pivotal to determine whether meclizine can be selected for a clinically beneficial alternative treatment.

## Materials and Methods

### Reagents and Antibodies

Meclizine dihydrochloride was obtained from Selleck Chemicals (Houston, TX, United States). The drug was dissolved in 10% Kolliphor^®^EL for *in vivo* use, and dissolved in DMSO for *in vitro* use. Recombinant soluble mouse M-CSF and RANKL were purchased from PeproTech (Rocky Hill, CT, United States). Rabbit antibody against NFATc1 (D15F1) was purchased from Cell Signaling Technology (Boston, MA, United States). Rabbit antibody against c-Fos (H-125) was obtained from Santa Cruz Biotechnology (Santa Cruz, CA, United States). Rabbit antibodies against p-ERK (D13.14.4E), ERK (137F5), p-p38 (D3F9), p38 (D13E1), p-JNK (81E11), JNK (#9252), p-IκBα (14D4), IκBα (L35A5), p-p65 (93H1) and p65 (D14E12) were purchased from Cell Signaling Technology. Rabbit anti-PXR (ab192579) was obtained from Abcam (Cambridge, MA, United States). Mouse anti-β-actin (8H10D10) was purchased from Boster (Wuhan, China).

### Animals

Female, 12 weeks old C57/BL6 female mice were obtained from the Experimental Animal Center of Tongji Hospital (Wuhan, China), fed in the animal care facility of Tongji Hospital. This animal study was authorized by the Ethics Committee on Animal Experimentation of Tongji Medical College (Wuhan, China).

Animals were divided randomly into four groups (*n* = 10 mice/group): sham-operated mice treated with 10% Kolliphor^®^ EL (vehicle) (SHAM), sham-operated mice treated with meclizine (20 mg/kg) (SHAM + meclizine), bilateral ovariectomized mice treated with vehicle (OVX) and bilateral ovariectomized mice were treated with meclizine (20 mg/kg) (OVX + meclizine) (Kishi et al., 2015). Meclizine solution or vehicle was injected intraperitoneally five times per week for 9 weeks. The body weight of mice was recorded every week and the mice were sacrificed to collect femurs, serum, and tibias for use in the following experiments after 9 weeks.

### Micro-Computed Tomography (μ-CT)

The distal femoral bone structure was analyzed by micro-computed tomography (μ-CT) system (μ-CT50 Scanco Medical, Bassersdorf, Switzerland) after removing soft tissues. Images were performed at 100 kV and 98 μA; the resolution was set to 10.5 μm. Three dimensional reconstruction were analyzed by the built-in software in the μ-CT system. Bone volume/tissue volume (BV/TV), trabecular number (Tb. N.), trabecular thickness (Tb. Th.), bone surface/trabecular bone volume (BS/BV), connectivity density (Conn. D), and trabecular separation (Tb. Sp.) were evaluated in the μ-CT system.

### Histomorphometric Analysis

For histomorphometric analysis, femur samples were put in 4% paraformaldehyde for 48 h, decalcified in 10% EDTA solution for 2 weeks and embedded in paraffin wax. The trabecular structure was observed by H&E staining. Tartrate-resistant acid phosphatase (TRAP) staining (Sigma–Aldrich, St. Louis, MO, United States) was performed following the standard protocols as well as the number of osteoclasts in the region was counted as previously described (Lee et al., 2006; Guan et al., 2015).

### Serum Biochemistry

For serum biochemical analysis, blood was gathered by retro-orbital puncture immediately before sacrifice. Sera were then extracted and serum levels of CTX-I were assessed with ELISA kits (IDS Nordic, Herlev, Denmark). Serum RANKL and OPG levels were evaluated by mouse RANKL and OPG ELISA kit (Boster).

### Cell Cultures

Bone marrow-derived macrophages (BMMs) were obtained from the femurs and tibias of 5-week-old C57BL/6 mice as described previously (Koga et al., 2004; Zhang Y. et al., 2015). Concisely, bone marrow cavities of isolated femurs and tibias were flushed with culture medium. Cells were then collected and cultured in α-MEM with 10% fetal bovine serum, 100 μg/mL streptomycin, 100 U/mL penicillin, and 30 ng/mL M-CSF. After 24 h, floating cells were gathered and supplemented with M-CSF (30 ng/mL). After 2 days, adherent cells were kept in different plates for use and cell density was given in the following methods.

### Cell Counting Kit-8 Assay

Cell proliferation and viability was assessed using CCK 8 assay (Boster). BMMs were seeded at density of 6,000 cells/well in 96-well plates. After 1 day, BMMs were added with DMSO (vehicle), 1, 5, 10, and 20 μM meclizine every day in the presence of 30 ng/mL M-CSF for 4 days. After 1, 2, 3, and 4 days, medium containing 10% CCK 8 solution was substituted for the culture medium and then the cells incubated in darkness at 37°C for an hour. The absorbance was then measured at 450 nm with ELX800 absorbance microplate reader (Bio-Tek, Winoosk, VI, United States).

### Actin Ring Formation Assays and DAPI Staining

For actin ring formation assays, BMMs (1.0 × 10^4^ cells/well) were cultured with different concentrations of meclizine for 4 days. Next, cells were added with immunol staining fix solution (Beyotime, Shanghai, China) for 10 min, then the cells were permeabilized with immunol staining wash buffer (Beyotime) for 5 min and incubated with phalloidin (Sigma–Aldrich, St. Louis, MO, United States) at 25°C for 30 min to visualize F-actin. After treatment with actin ring staining, cells were washed four times with phosphate buffer saline followed by staining with DAPI (Boster) for 5 min. Images were obtained using fluorescence microscope.

### *In Vitro* Osteoclastogenesis Assay

Bone marrow-derived macrophages were seeded at a density of 10,000 cells/well in 96-well plates and cultured with M-CSF (30 ng/mL) and RANKL (100 ng/mL) in the presence of vehicle (DMSO) or meclizine. We replaced the culture medium every day. Osteoclasts were stained by TRAP staining kit according to the manufacturer’s protocol after 4 days. TRAP-positive multinucleated (≥3 nuclei) cells were counted as osteoclasts (Asagiri and Takayanagi, 2007). Images were obtained using EVOS FL auto cell image system (Life Technologies, paisley, United Kingdom).

### Bone Pit Formation by Osteoclasts

We performed pit formation assay as described previously (Guan et al., 2015). Briefly, BMMs were seeded in a Corning Osteo Assay Surface plate (Corning Incorporated Life Science, Corning, NY, United States) at a density of 20,000 cells/well. Next, cells were treated with M-CSF (30 ng/mL) and RANKL (100 ng/mL) for 4 days, then treated with vehicle (DMSO) or different concentrations of meclizine. After additional 4 days, the plate was washed with 5% sodium hypochlorite for 5 min and PBS for 10 min. Images of bone resorption were obtained and quantified.

### RNA Extraction, Reverse Transcription and Quantitative RT-PCR

Quantitative RT-PCR was used as described previously (Zhang Y. et al., 2015). In brief, BMMs with a density of 20,000 were plated in each well of a 24-well plate, cultured with M-CSF (30 ng/mL) and RANKL (100 ng/mL) in the presence of vehicle (DMSO) or meclizine. Total RNA was extracted from BMMs using TRIzol reagent (Invitrogen Life Technologies, Carlsbad, CA, United States). cDNA was synthesized using ReverTra Ace qPCR RT Kit (Toyobo, Osaka, Japan), then we performed qRT-PCR using the THUNKERBIRD SYBR qPCR Mix (Toyobo) and Bio Rad Q5 instrument (Bio Rad Laboratories, Hercules, CA, United States). All processes were applied according to the manufacturer’s protocol, and the expression of target genes was normalized to the reference gene *GAPDH*. The following primers were used (F, forward; R, reverse):

*NFATc1*, F 5′-GACCCGGAGTTCGACTTCG-3′ and R 5′-TGACACTAGGGGACAC ATAACTG-3′; *TRAP*, F 5′-CACTCCCACCCTGAGATTTGT-3′ and R 5′-CATCGTCTGCACGG TTCTG-3′; *CK*, F 5′-GAAGAAGACTCACCAGAAGCAG-3′ and R 5′-TCCAGGTTATGGGCA GAGATT-3′; *MMP9*, F 5′-CTGGACAGCCAGACACTAAAG-3′ and R 5′-CTCGCGGCAAGTCT TCAGAG-3′; *GAPDH*, F 5′-ACCCAGAAGACTGTGGATGG-3′ and R 5′-CACATTGGGGGTAG GAACAC-3′.

### Western Blot Analysis

Western blotting was performed as previously described (Dong et al., 2016, 2017). Concisely, cells were treated with the RIPA Lysis Buffer (Boster) containing protease inhibitor cocktail (Boster). The concentration of total cell proteins was measured by the BCA assay (Boster). Equivalent quality of proteins was added in 10% SDS-polyacrylamide gel and transferred to PVDF membranes (Millipore, Billerica, MA, United States). After blocking with 5% bovine serum albumin, membranes were incubated overnight with corresponding antibodies at 4°C. Subsequently, the blot was washed and incubated with horseradish peroxidase -conjugated secondary antibodies (Boster) for 1h at room temperature. The immunoreactive proteins were determinated with enhanced chemiluminescence (Boster) and images were obtained by ChemiDoc^TM^ XRS + System with Image Lab^TM^ Software (Bio-Rad Laboratories).

### Small Interfering RNA (siRNA) Assays

BMMs, seeded at a density of 10,000 cells/well in 96-well plates, were transfected with siRNAs (20 μM) (Ribbbio, Guangzhou, China) using siRNA Transfection Reagent (Ribbio) with M-CSF (30 ng/mL) according to the manufacturer’s protocol. After incubating with the siRNA mix (100 nM) for 5 h, the cells were added with culture medium in the presence of RANKL (100 ng/mL) and incubated for 2 days. Then, the transfection was repeated. On day 4, multinucleated osteoclasts were stained with a TRAP staining kit. Cell lysates were collected for further experiment.

### Statistical Analysis

All experiments were independently performed at least three times. Results were expressed as means ± standard difference (*SD*). The student’s *t*-test was used for statistical analysis between two groups and statistical comparison of multiple comparisons was performed using ANOVA followed by a Tukey test. ^∗^*p* < 0.05 and ^∗∗^*p* < 0.01 indicated significantly difference.

## Author Contributions

FG, JX, JG, and YW conceived and designed the study. JG, WL, and XJ performed the experiment. JH, RR, JG, and JZ analyzed the data. FG, JX, and JG wrote the paper. ZL and WX revised the manuscript. All authors have contributed to the final version and approved the publication of the final manuscript.

## Conflict of Interest Statement

The authors declare that the research was conducted in the absence of any commercial or financial relationships that could be construed as a potential conflict of interest.
