# Corrigendum: Meclizine Prevents Ovariectomy-Induced Bone Loss and Inhibits Osteoclastogenesis Partially by Upregulating PXR

**DOI:** 10.3389/fphar.2017.00991

**Published:** 2018-01-11

**Authors:** Jiachao Guo, Weijin Li, Yingxing Wu, Xingzhi Jing, Junming Huang, Jiaming Zhang, Wei Xiang, Ranyue Ren, Zhengtao Lv, Jun Xiao, Fengjing Guo

**Affiliations:** ^1^Department of Orthopedics, Tongji Hospital, Tongji Medical College, Huazhong University of Science and Technology, Wuhan, China; ^2^Department of Otolaryngology-Head and Neck Surgery, Tongji Hospital, Tongji Medical College, Huazhong University of Science and Technology, Wuhan, China

**Keywords:** meclizine, PXR, osteoclast, RANKL, osteoporosis

There is an error in the Funding statement. The correct number for 81372915 is 81371915. The authors apologize for this error and state that this does not change the scientific conclusions of the article in any way.

The original article has been updated.

## Conflict of interest statement

The authors declare that the research was conducted in the absence of any commercial or financial relationships that could be construed as a potential conflict of interest.

