# Supplementary material for: Meclizine Prevents Ovariectomy-Induced Bone Loss and Inhibits Osteoclastogenesis Partially by Upregulating PXR
Source: Front Pharmacol. 2017 Oct 4;8:693. doi: 10.3389/fphar.2017.00693 (PMC5632684; doi:10.3389/fphar.2017.00693)
Supplement: Supplementary file 1 [file Image_1.PDF]

Supplementary Material

Meclizine Prevents Ovariectomy-Induced Bone Loss and Inhibits Osteoclastogenesis Partially by Upregulating PXR

Jiachao Guo, Weijin Li, Yingxing Wu, Xingzhi Jing, Junming Huang, Jiaming Zhang, Wei Xiang, Ranyue Ren, Zhengtao Lv, Jun Xiao* and Fengjing Guo*

* Correspondence:
Prof. Jun Xiao Prof. Fengjing Guo
jun_xiao@hust.edu.cn; guofjdoc@163.com
Tel.: +86-27-8366-5238 (J.X.); +86-27-8366-5218 (F.G.)
Fax: +86-27-8366-3770 (J.X.); +86-27-8366-3670 (F.G.)

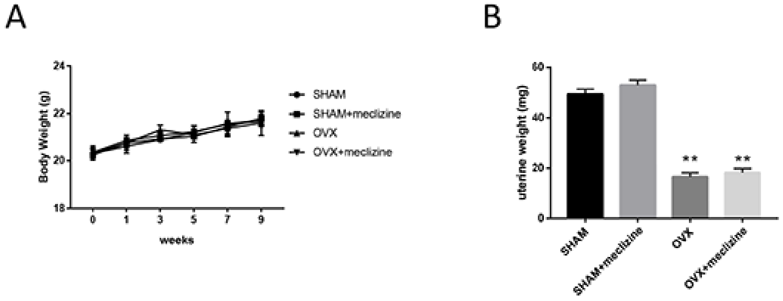

Supplementary Figure 1. (A) The body weight of mice was recorded every 2 weeks. Data are presented as means ± SD. n=10. **(B)** Mice were sacrificed after 9 weeks of meclizine treatment. Mice uterus was isolated and weighed. Data are presented as means ± SD. n=10. **P<0.01 versus SHAM group.
